# Prevalence and Significance of Pyuria in Chronic Kidney Disease Patients in Saudi Arabia

**DOI:** 10.3390/jpm11090831

**Published:** 2021-08-25

**Authors:** Lina Almaiman, Khaled S. Allemailem, Asmaa M. El-Kady, Mishaal Alrasheed, Ahmad Almatroudi, Fahad S. Alekezem, Abdelrahman Elrasheedy, Wafa Abdullah Al-Megrin, Hussah M. Alobaid, Hatem A. Elshabrawy

**Affiliations:** 1 Department of Medical Laboratories, College of Applied Medical Sciences, Qassim University, Buraydah 51452, Saudi Arabia; lenaalmaiman@gmail.com (L.A.); k.allemailem@qu.edu.sa (K.S.A.); aamtrody@qu.edu.sa (A.A.); 2 Department of Medical Parasitology, Faculty of Medicine, South Valley University, Qena 83523, Egypt; asmaa.elkady@med.svu.edu.eg; 3 Department of Laboratory and Blood Bank, King Fahd Specialist Hospital, Buraydah 52211, Saudi Arabia; hha_443@hotmail.com (M.A.); momi010@hotmail.com (F.S.A.); abdelrahmanelrasheedy@gmail.com (A.E.); 4 Department of Biology, Faculty of Science, Princess Nourah bint Abdulrahman University, Riyadh 11671, Saudi Arabia; waalmegrin@pnu.edu.sa; 5 Department of Zoology, College of Science, King Saud University, Riyadh 11362, Saudi Arabia; hesalobaid@ksu.edu.sa; 6 Department of Molecular and Cellular Biology, College of Osteopathic Medicine, Sam Houston State University, Conroe, TX 77304, USA

**Keywords:** CKD, pyuria, UTI, *E. coli*, WBCs, urine, leukocyte esterase, albuminuria, urinary nitrite, bacteria

## Abstract

Chronic kidney disease (CKD) is considered a major health problem, which poses a burden for health care systems worldwide. It has been estimated that 10% of the population worldwide have CKD; however, most of the cases are undiagnosed. If left untreated, CKD could lead to kidney failure, which highlights the importance of early diagnosis and treatment. Pyuria has been reported in CKD patients, and could be the result of several comorbidities, such as diabetes, or urinary tract infections (UTIs). A few studies have shown that pyuria is associated with the late stages of CKD. However, there are limited data on the prevalence of non-UTI (sterile) and UTI–pyuria in different CKD patient populations, and its association with the decline in kidney function and progression of CKD. In this retrospective study, we report the prevalence of pyuria (sterile and UTI) in 754 CKD patients of King Fahd Specialist Hospital, Buraydah, Saudi Arabia. Our data showed that 164/754 CKD patients (21.8%) had pyuria, whereas 590 patients (78.2%) presented with no pyuria. There was a significantly higher percentage of late-stage (stage 4) CKD patients in the pyuric group compared to the non-pyuric group (36.6% vs. 11.9%). In line with the previous data, proteinuria was detected in a significantly higher percentage of pyuric patients, in addition to significantly higher levels of serum creatinine and urea, compared to non-pyuric patients. Furthermore, 13.4% of the pyuric CKD patients had UTI, whereas 86.6% presented with sterile pyuria. *E. coli* was indicated as the causative agent in 45.5% of UTI patients. Our patient data analysis showed that a significantly higher percentage of UTI–pyuric CKD patients, than sterile pyuric patients (63.6% vs. 19.7%), had higher numbers of urinary white blood cells (>50/HPF, WBCs). The data also showed that a higher percentage of UTI–pyuric patients were late-stage CKD patients, compared to sterile pyuric patients (50% vs. 34.5%). Our findings indicate that a high level of pyuria could be considered as a marker for late-stage CKD, and that UTI is an important risk factor for the decline in kidney function and the progression to late-stage CKD. We believe that further studies are needed to correlate pyuria to kidney function, which could be helpful in monitoring the progression of CKD. Moreover, the management of comorbidities, such as diabetes and UTIs, which are risk factors for CKD and associated pyuria, could help to control the progression of CKD to the late stages.

## 1. Introduction

Chronic kidney disease (CKD) is one of the leading causes of death in the United States [1]. Several studies, in Africa, Asia, Australia, Europe, and South and North America, have also shown the high burden of this disease in several countries [2]. In Saudi Arabia, the prevalence of CKD is around 5.7% and it is considered the fourth leading cause of death, with a mortality rate of 5.44% [3].

CKD could be a risk factor for urinary tract infections (UTIs), which can manifest as asymptomatic bacteriuria or symptomatic UTIs that require treatment [4]. It is believed that CKD compromises innate and adaptive immune responses, which renders CKD patients more susceptible to infections, including UTIs [5]. Diabetes, obstructions in the urinary tract, and old age, are known to increase the risk of UTI, CKD, and its progression to the late stages [6]. If left undiagnosed or untreated, UTI could result in significant deterioration in renal function, advanced stages of CKD, nephrectomy, and death [7,8,9].

Pyuria is a useful marker for UTIs in the general population, especially in symptomatic patients; however, pyuria may also present in the absence of UTIs, such as in the cases of structural abnormalities of the genitourinary tract or interstitial nephritis [4,10]. Pyuria has also been described in CKD patients, in the presence and in the absence of UTIs (sterile pyuria) [10,11]. To the best of our knowledge, sterile pyuria is more prevalent in the CKD patient population than in the non-CKD population, and it positively correlated with CKD stage [10,11]. Studies have shown that the prevalence of sterile pyuria among CKD patients was in the range of 31–72% [12,13,14,15,16,17]. This may be explained by chronic renal parenchymal inflammation, due to comorbidities such as diabetes and hypoalbuminemia [12,13]. Furthermore, 25–45% of pyuric CKD patients could also present with UTI, which could be a complication of CKD, or comorbidities such as diabetes [4,6,12,16,18]. It has been reported that UTI–CKD patients had high levels of pyuria and that pyuria associated with advanced stages of CKD [4,10]. However, the association between pyuria and the decline in kidney function needs further investigation.

Despite all the previous studies, there are limited studies on the prevalence of sterile and UTI pyuria among CKD patients of different populations. In the present study, we aimed to study the prevalence of sterile and UTI pyuria in CKD patients of the King Fahd Specialist Hospital, Buraydah, Qassim, Saudi Arabia, in the period from March to December 2020. We also attempted to assess the association between pyuria and the late stages of CKD.

## 2. Materials and Methods

### 2.1. Study Population

The present study is a single-center, retrospective, cross-sectional study that was conducted at the King Fahd Specialist Hospital, Buraydah, Qassim, Saudi Arabia. All CKD patients (754 patients) who attended the outpatient nephrology clinic in the period from March to December 2020, were included in the study. CKD was classified into stage I (kidney damage with normal or increased kidney function) with glomerular filtration rate (GFR) ≥ 90, stage II (kidney damage with mildly diminished kidney function) with GFR between 60 and 89, stage III (moderately reduced kidney function) with GFR between 30 and 59, stage IV (severely decreased kidney function) with GFR between 15 and 29, and stage V is considered as (kidney failure) with GFR < 15 [19].

Demographic data (age, gender, stage of CKD and comorbidities such as diabetes mellitus, hypertension, chronic pulmonary diseases, malignancy, chronic organ failure, etc.) and laboratory results (including complete urine analysis (presence of red blood cells and white blood cells in urine sediment, proteinuria, presence of nitrite, leukocyte esterase), kidney function tests, hemoglobin, and urine culture) were retrieved from the electronic patients’ records.

Pyuria was defined as ≥5 WBCs per high-power field (HPF) of centrifuged urine examined by microscopy, which is still considered the standard method [20]. Hematuria was defined as ≥5 red blood cells/HPF of urine examined by microscopy. Proteinuria was defined as the presence of ≥1+ protein in urine using dipstick analysis. Presence of urinary nitrites and urinary leukocyte esterase were determined using urine dipstick analysis. Pyuria with significant bacteriuria (≥10^5^ colony forming units/mL (CFU/mL)) was defined as UTI, whereas pyuria without significant bacteriuria (<10^5^ CFU/mL) was defined as sterile pyuria.

### 2.2. Statistical Analysis

Data analysis was carried out using the IBM SPSS 20.0 software (SPSS Inc., Chicago, IL, USA). Continuous variables were expressed as mean ± standard deviation (SD). Categorical data were expressed as numbers and percentages. Statistical analyses were performed using Mann–Whitney U test or chi-square test for continuous and categorical variables, respectively. The *p* values < 0.05 were considered statistically significant.

## 3. Results

### 3.1. Patients’ Characteristics

**Figure 1:**
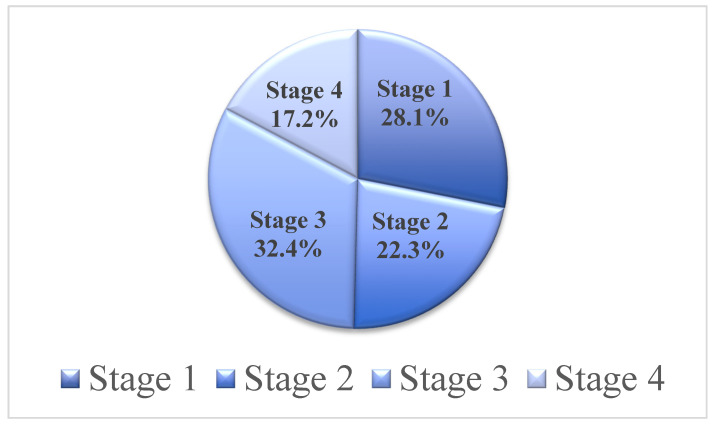
Categorization of patients according to stage of chronic kidney disease (CKD). Most of patients were diagnosed as stage 3 CKD patients (33%) followed by stage 1 (28%), stage 2 (22%), and stage 4 (17%).

Seven hundred and fifty-four (754) CKD patients, included in the present study, were categorized according to the CKD stage, into stage 3 (32.4%), stage 1 (28.1%), stage 2 (22.3%), and stage 4 CKD patients (17.2%) (Figure 1). The age of the patients ranged from 14 to 95 years (mean ± SD = 56 ± 17), with a majority being males (62.6%). The medical histories showed that 74.8% of the CKD patients are hypertensive, whereas 57.4% have diabetes. Moreover, urine analysis revealed that 48.5% of the patients had albuminuria upon dipstick examination, 2.9% had urinary tract infection, 20.3% showed hematuria, 2.3% were positive for nitrite, whereas 16.6% were positive for leukocyte esterase (Table 1). The serum urea and creatinine were higher than normal, which correlates with the CKD condition of these patients.

### 3.2. Pyuric CKD Patients Are in Later Stages of CKD and Could Present with UTI Compared to Non-Pyuric Patients

Our results show that 164 CKD patients (21.8%) had pyuria, with a significantly higher percentage of females (68.9% vs. 28.6%) and a lower percentage of males (31.1% vs. 71.4%), compared to the non-pyuric CKD patients (Table 2). There was no significant difference between the mean age of the pyuric and non-pyuric CKD patients. However, there was a significantly higher percentage of late-stage (stage 4) CKD patients in the pyuric group compared to the non-pyuric group of patients (36.6% vs. 11.9%; *p* = 0.002). This finding indicates that the existence of pyuria in CKD patients could be diagnostic for the late stages of CKD.

Next, we compared the clinical and laboratory characteristics of pyuric and non-pyuric CKD patients. Our results showed that significantly higher percentages of pyuric, than non-pyuric, CKD patients, had proteinuria (albuminuria), *p* = 0.001. The prevalence of proteinuria (64%), and the significantly higher levels of serum urea (*p* = 0.006) and creatinine (*p* = 0.001) in the pyuric patients, compared to the non-pyuric patients, correlates with higher percentage of late-stage CKD patients in the pyuric group compared to the non-pyuric group. Consistent with the existence of pyuria, a significantly higher percentage of pyuric patients tested positive for urinary leukocyte esterase compared to non-pyuric patients (67.7% vs. 2.4%; *p* = 0.002). Unlike the non-pyuric patients, who presented with no UTI, 13.4% of the pyuric patients had UTI, which resulted in a significantly higher percentage of pyuric patients testing positive for urinary nitrite compared to non-pyuric patients (7.9% vs. 0.7%; *p* = 0.002). Our data report a significantly higher percentage of hematuric pyuric patients than hematuric non-pyuric patients (43.9% vs. 13.7%; *p* = 0.004), which indicates that pyuria could be associated with hematuria. Additionally, we found that comorbidities, such as diabetes and hypertension, were more prevalent in the non-pyuric group than in the pyuric group (Table 2).

### 3.3. Several Bacterial Species Were Associated with UTI in 22 Pyuric CKD Patients with E. coli Being the Most Common Causative Agent

As previously shown in Table 2, 22 out of our 164 pyuric CKD patients (13.4%) were diagnosed with UTI. The most common causative agent of UTI in our patients was *Escherichia coli* (*E. coli*) (10 out of 22 (45.5%) UTI patients). Several other microorganisms were reported as UTI causative agents (Table 3).

### 3.4. Pyuric CKD Patients Diagnosed with UTI Have Increased Numbers of Urinary WBCs and Are in Later Stages of CKD Compared to Sterile Pyuric CKD Patients

As previously mentioned, 13.4% (22 patients) of pyuric CKD patients had UTI, whereas 86.6% (142 patients) were sterile pyuric patients. Next, we compared the demographics and various clinical parameters of sterile pyuric and UTI–pyuric CKD patients (Table 4). There were no significant differences in the mean age (*p* = 0.095) and gender distribution (*p* = 0.063) between the sterile and UTI–pyuric patients. As expected, a significantly higher percentage of UTI–pyuric patients had a high number (>50/HPF) of urinary white blood cells (WBCs) compared to sterile pyuric patients (63.6% vs. 19.7%; *p* = 0.002). Furthermore, a significantly higher percentage of UTI–pyuric patients than sterile pyuric patients had an advanced stage of CKD (50% vs. 34.5%; *p* = 0.004). Interestingly, the sterile pyuric group had a significantly higher percentage of patients with late-stage CKD than non-pyuric patients (34% vs. 11.9%; *p* < 0.05) (Table 2 and Table 3). The previous findings confirm that a high level of pyuria is a significant indicator of late-stage CKD. The significantly higher percentage of urinary leukocyte esterase-positive UTI–pyuric patients (86.4%; *p* = 0.002) confirmed our finding of a higher number of urinary WBCs in a significant percentage of these patients. Moreover, the significantly higher percentage of urinary nitrite-positive patients among the UTI–pyuric patients, compared to the sterile pyuric patients (18.2% vs. 6.3%; *p* = 0.003), confirmed the infection status of these patients. There were no significant differences in the prevalence of diabetes, hypertension, and proteinuria, or the levels of serum urea, creatinine, and hemoglobin, between the UTI and sterile pyuric CKD patients (*p* > 0.05). The nonsignificant difference in the percentage of proteinuria patients, and levels of serum urea and creatinine, between the UTI–pyuric and sterile pyuric patients, despite the higher percentage of late-stage CKD patients in the UTI–pyuric group, may be due to a smaller number of UTI–pyuric patients compared to sterile pyuric patients (22 vs. 142). Overall, our findings indicate that UTI is an important risk factor for the late stages of CKD, and these late stages of CKD could be predicted by detecting high numbers of urinary WBCs (>50/HPF; high pyuria).

## 4. Discussion

CKD is a growing health care issue that is associated with the expenditure of millions of dollars, resulting in a huge burden on the economy and health care systems [21,22]. According to CDC, the 2018 Medicare spending on CKD patients was USD 81.8 billion or USD 23,700 per person. It has been estimated that 37 million adults in the United States (US) have CKD, of which most cases are undiagnosed. Diabetes and high blood pressure are important risk factors for CKD [23].

Pyuria (the presence of WBCs in urine) is a common presentation in CKD patients, and could exist in the presence or absence of UTI (sterile pyuria) [10,11]. Pyuria in CKD patients is believed to be the result of renal parenchyma inflammation, which could be exacerbated by advanced age, female gender, diabetes, hypoalbuminemia, and UTI [4]. The existence of UTI in some CKD patients could be the deleterious effect of comorbidities, such as diabetes, urinary tract obstruction, and advanced age, all of which increase the risk of UTI [6]. However, CKD itself is believed to be a risk factor for UTI, due to impairment of cellular and humoral immunity [24]. Despite the prevalence of pyuria in CKD patients, the association between pyuria and the deterioration of kidney function in CKD patients is not well understood.

In the present study, our data showed that 164 CKD patients (21.8%) had pyuria. This is in agreement with previous reports, which showed the prevalence of pyuria (31–72%) among CKD patients [12,13,14,15,16,17]. Furthermore, our results showed that a significantly higher percentage of CKD pyuric patients, than non-pyuric CKD patients, were late-stage CKD patients (stage 4). These results are in agreement with studies that showed that 17.5% of late-stage CKD patients had pyuria, and that pyuria is an indicator of the deterioration of kidney function in patients with autosomal dominant polycystic kidney disease (ADPKD) [4,8].

Sterile pyuria was more predominant in our CKD patients compared to UTI-associated pyuria (142 sterile pyuric patients (86.6%) vs. 22 UTI pyuric patients (13.4%)). Few studies reported the prevalence of UTI among CKD patients; however, the authors suggested that the frequency of UTI is quite similar to that in the general population [24]. Our data showed that a significantly higher percentage of UTI, than sterile pyuric patients (63.6% vs. 19.7%), had higher pyuria (>50 WBCs/HPF). This finding is consistent with another study, which showed that the degree of pyuria was associated with UTI in CKD patients [10]. In addition, a significantly higher percentage of UTI, than sterile pyuric CKD patients, were late-stage CKD patients (50% vs. 34.5%). Other studies have shown that UTI may result in acute kidney injury (AKI) and, eventually, CKD [25]. Several mechanisms that are involved in UTI-mediated kidney injury have been described, including renal parenchyma infection and sepsis [26,27]. Our findings indicate that pyuria is an important indicator of the decline in kidney function in CKD patients, which is supported by a previous study that concluded that pyuria precedes UTI and the subsequent deterioration of kidney function in ADPKD patients [8]. Furthermore, our findings support the need for the early management of UTIs and other comorbidities in CKD patients, to prevent the decline in kidney function, and indicates that pyuria could be used to monitor the progression of CKD.

## Figures and Tables

**Table 1:** Demographic, clinical and laboratory data of chronic kidney disease (CKD) patients. CKD patients’ demographic and clinical data are shown and expressed as numbers (*N*) and percentages (%) or as mean ± SD.

Patients’ Characteristics		*N* = 754 56 ± 17	
Age (Years; Mean ± SD)			
		*N*	%
**Gender**	Male	472	62.6
	Female	282	37.4
**CKD Stage**	Stage 1	212	28.1
	Stage 2	168	22.3
	Stage 3	244	32.4
	Stage 4	130	17.2
**Diabetes**		433	57.4
**Hypertension**		564	74.8
**Albuminuria**		366	48.5
**Hematuria**		153	20.3
**Urinary Tract Infection (UTI)**		22	2.9
**Urinary Leukocyte Esterase**		125	16.6
**Urinary Nitrite**		17	2.3
		**Mean ± SD**	
**Serum Urea (mmol/L)**		10.8 ± 7.9	
**Serum Creatinine (μmol/L)**		192.5 ± 146.2	
**Hemoglobin (g/dL)**		13.02± 2.23	

**Table 2:** Characteristics of pyuric and non-pyuric CKD patients. Demographic, clinical, and laboratory data of pyuric (*N* = 590) and non-pyuric patients (*N* = 164) are presented as numbers (*N*) and percentages (%) or means ± SD. The *p*-values < 0.05 were considered statistically significant.

Variables		No Pyuria *N* = 590		Pyuria *N* = 164		*p*-Value
**Age** (years; mean ± SD)		56.2 ± 16		53.2 ± 19.5		0.151
		***N***	**%**	***N***	**%**	
**Gender**	Male	421	71.4	51	31.1	0.001
	Female	169	28.6	113	68.9	
**CKD Stage**	Stage 1	183	31	29	17.7	0.002
	Stage 2	137	23.2	31	18.9	
	Stage 3	200	33.9	44	26.8	
	Stage 4	70	11.9	60	36.6	
**Diabetes**		353	59.8	80	48.8	0.008
**Hypertension**		455	77.1	109	66.5	0.004
**Albuminuria**		261	44.2	105	64	0.001
**Hematuria**		81	13.7	72	43.9	0.004
**Urinary Tract Infection (UTI)**		0	0	22	13.4	0.003
**Urinary Leukocyte Esterase**		14	2.4	111	67.7	0.002
**Urinary Nitrite**		4	0.7	13	7.9	0.002
		**Mean ± SD**				
**Serum Urea (mmol/L)**		10 ± 6.7		13.8 ± 10.7		0.006
**Serum Creatinine (μmol/L)**		175.9 ± 136.9		252.5 ± 228.7		0.001
**Hemoglobin (g/dL)**		13.32 ± 2.209		11.98 ± 1.963		0.001

**Table 3:** *Escherichia coli* (*E. coli*) is the most common causative agent of urinary tract infections (UTIs) in pyuric CKD patients. The microbial causative agent associated with each UTI case was retrieved from patients’ electronic medical records. The number of patients infected with each microbial species was recorded.

Organism	Number of Patients
*Acinetobacter lwoffii*	1
*Candida Species*	1
*Citrobacter freundii*	1
*E. coli*	10
*Staphylococcus aureus*	1
*Enterococcus fecalis*	2
*Klebsiella pneumoniae*	3
*Pseudomonas Aeruginosa*	2
*Salmonella Species*	1
Total	22

**Table 4:** Comparison of demographic and clinical parameters of UTI and sterile pyuric CKD patients. Demographic, clinical, and laboratory data of UTI–pyuric (*N* = 22) and sterile pyuric patients (*N* = 142) were collected and presented as numbers (*N*) and percentages (%) or means ± SD. The *p*-values < 0.05 were considered statistically significant.

Variables		UTI		Sterile Pyuria		*p*-Value
		*N* = 22		*N* = 142		
**Age** (years; mean ± SD)		55 ± 22		53 ± 19		0.095
		***N***	**%**	***N***	**%**	
**Gender**	Males	7	31.8	44	31	0.063
	Females	15	68.2	98	69	
**CKD Stage**	Stage 1	2	9.1	27	19.1	0.004
	Stage 2	3	13.6	28	19.9	
	Stage 3	6	27.3	38	27	
	Stage 4	11	50	49	34.5	
**Urinary WBCs/HPF**	5–9	4	18.2	44	31	0.002
	10–19	3	13.6	39	27.5	
	20–49	1	4.6	31	21.8	
	>50	14	63.6	28	19.7	
**Diabetes**		12	54.5	68	44.7	0.6
**Hypertension**		13	59.1	96	67.6	0.44
**Albuminuria**		14	63.6	91	64.1	0.353
**Hematuria**		8	36.4	64	45.1	0.037
**Urinary Nitrite**		4	18.2	9	6.3	0.003
**Urinary Leukocyte Esterase**		19	86.4	92	64.8	0.002
		**Mean ± SD**				
**Serum Urea (mmol/L)**		17.1 ± 10.4		13.3 ± 10.7		0.072
**Serum Creatinine (μmol/L)**		269.05 ± 206.62		250.08 ± 232.49		0.061
**Hemoglobin (g/dL)**		13.08 ± 2.212		11.27 ± 2.021		0.121

## Data Availability

Not applicable.
